# Renoprotective effect of topiroxostat via antioxidant activity in puromycin aminonucleoside nephrosis rats

**DOI:** 10.14814/phy2.13358

**Published:** 2017-08-03

**Authors:** Yosuke Kawamorita, Takeshi Shiraishi, Yoshifuru Tamura, Takanori Kumagai, Shigeru Shibata, Yoshihide Fujigaki, Makoto Hosoyamada, Takahiko Nakagawa, Shunya Uchida

**Affiliations:** ^1^ Department of Internal Medicine Teikyo University School of Medicine Tokyo Japan; ^2^ Support for Community Medicine Endowed Chair Teikyo University School of Medicine Tokyo Japan; ^3^ Department of Human Physiology and Pathology Faculty of Pharma‐Sciences Teikyo University Tokyo Japan; ^4^ Industry‐Academia‐Government Association Promotion Center Nara Medical University Nara Japan

**Keywords:** Cell uric acid, NOX4, puromycin aminonucleoside nephrosis, topiroxostat

## Abstract

Topiroxostat is a novel inhibitor of xanthine oxidase, and is postulated to exert a renoprotective effect. Puromycin aminonucleoside nephrosis (PAN) is a rat model of minimal change nephrotic syndrome. In this study, we examined whether topiroxostat ameliorates the kidney injury in PAN rats that was induced by a single intraperitoneal injection of PA (100 mg/kg body weight). Rats were divided into four groups: control rats, PAN rats, control rats treated with topiroxostat (1.0 mg/kg/day), and PAN rats treated with topiroxostat. Topiroxostat significantly reduced the amount of uric acid in the kidney cortex, while serum UA concentration remained unaffected by this treatment. Urinary protein excretion decreased significantly on day 10 in PAN rats upon topiroxostat treatment. Podocyte injury in PAN rats, as indicated by the reduction in WT‐1‐positive cell numbers and podocin immunoreactivity and foot process effacement, was partially yet significantly alleviated with topiroxostat treatment. In the kidney cortex, the increase in oxidative stress markers such as nitrotyrosine and 8‐hydroxy‐2‐deoxyguanosine (8‐OHdG) and the enhanced expressions of xanthine oxidase and NADPH oxidase 4 (NOX4) in PAN rats were significantly ameliorated by topiroxostat. Using cultured podocytes NOX4 expression was upregulated by adding 12 mg/dL UA into the culture medium. These results suggest that topiroxostat ameliorates proteinuria and kidney injury in PAN rats by lowering oxidative stress and tissue UA concentration. The renoprotective effects of topiroxostat could be attributed to its potential to inhibit xanthine oxidase and NOX4 in concert with suppression of intracellular UA production.

## Introduction

Accumulating evidence suggests that uric acid (UA) aggravates kidney diseases and vascular injury. Mechanisms whereby UA deteriorates kidney function may be ascribed to the renal vasculopathy and hypertension resulting from the thickening of afferent artery and tubulointerstitial injury due to activation of renin‐angiotensin system, inflammasome, and microinflammation (Kang et al. 2002). Several studies have suggested that inhibition of UA production by a xanthine oxidase (XO) inhibitor, allopurinol, is a potential therapeutic option in the treatment and management of a variety of kidney diseases (Kang et al. 2002; Nakagawa et al. 2003; Feig et al. 2008; Kosugi et al. 2009).

Puromycin aminonucleoside (PA) is known to induce massive proteinuria transiently that naturally resolves over time. It is said that puromycin aminonucleoside nephrosis (PAN) rats is a rat model of minimal change nephrotic disease because vast areas of foot process effacement take place without marked histological abnormality on light microscopy. Of interest is that allopurinol reduced urinary protein excretion in PAN rats (Diamond et al. 1986). In this model, the effects of allopurinol on decrease in proteinuria include a reduction in oxidative stress via XO inhibition. Moreover, allopurinol exerted renoprotective effect with a reduction in urinary albumin excretion in a db/db mouse model of type 2 diabetes (Kosugi et al. 2009; Nakamura et al. 2016). Notably, serum UA concentration, which is extremely low at baseline because of the presence of uricase in rats, did not significantly decrease in response to allopurinol treatment and allopurinol reduced urinary protein excretion independently of lowering serum UA (Mazzali et al. 2001). In addition, human studies investigating the effects of allopurinol on urinary protein excretion are scarce, leading to incomplete understanding of the renoprotective effect of allopurinol and the role of XO inhibition in such effects.

Topiroxostat, a novel XO inhibitor, is attracting increasing attention because of its potential to decrease urinary albumin excretion in hyperuricemic patients with stage 3 chronic kidney disease (CKD) (Hosoya et al. 2014). However, the precise mechanisms remain unknown. Here, we examined whether topiroxostat exhibits renoprotective effects in the PAN rats and investigated the underlying molecular mechanisms with a focus on oxidative stress and its inhibition in this model rat.

## Materials and Methods

### Experimental design

All animal experiments were performed in accordance with the Institutional Animal Care and Use Committee of the Teikyo University, School of Medicine (Animal Ethics Committee, No. 14‐016). Male Sprague–Dawley rats (150–200 g body weight) underwent kidney function assessments and were randomly assigned to four groups: (1) control group (CONT group); (2) PAN group; (3) topiroxostat treatment group (Tx group); and (4) PAN with topiroxostat treatment group (PAN + Tx group). PAN group received a single intraperitoneal injection of PA (100 mg/kg body weight; Sigma, Saint Louis, MO). The rats in Tx and PAN + Tx groups received 1 mg/kg/day of topiroxostat in chow (10 mg/kg base chow; D10001, Research Diets, Inc. New Brunswick, NJ) (American‐Institute‐of‐Nutrition, 1977) from the following day. The animals were allowed free access to water and chow. On days 0 and 10, the rats were placed in metabolic cages and 24‐h urine samples were collected for measurement of urinary protein and creatinine concentrations. Blood samples were also collected on days 0 and 10. All the rats were killed on day 10 and their kidneys were removed, then the cortical tissues were isolated by carving from the capsule, snap frozen in liquid N_2_, and stored at −80°C until use.

### Laboratory studies

Urine protein content was measured using a protein assay reagent (Thermo Fisher Scientific, Waltham, MA). Serum and urine creatinine, total serum cholesterol, serum albumin, and serum UA concentrations were enzymatically determined using commercial kits (Oriental Yeast, Tokyo, Japan). UA concentration in kidney cortex was measured by a direct method using a commercial kit (QuantiChrom, BioAssay Systems, Hayward, CA) and expressed as mg/mg protein. Serum hypoxanthine and xanthine were measured using LC–MS method (LTQ‐Orbitrap, Thermo Fisher Scientific, Waltham, MA).

### Periodic acid–Schiff stain

Methyl Carnoy's solution‐fixed, paraffin‐embedded sections (1 *μ*m) were stained with the periodic acid‐Schiff reagent for light microscopy. The kidneys from all rats were histologically examined. To measure the glomerular size, coronal sections of each kidney were scanned using NanoZoomer (Hamamatsu Photonics, Shizuoka, Japan). Tuft areas of all glomeruli in each coronal section were traced using imaging software (Aperio Technologies, Vista, CA) to measure the glomerular size. Kidney sections were observed by two investigators in a blinded manner.

### Primary antibodies for immunohistochemistry and western blotting

Goat anti‐human type IV collagen antibody (Southern Biotech, Birmingham, AL) was used for the assessment of glomerular injury. Rabbit anti‐human NPHS2 (podocin) antibody (Abcam, Cambridge, MA) and rabbit anti‐human WT‐1 antibody (Santa Cruz Biotechnology, Santa Cruz, CA) were used to detect podocytes. Mouse anti‐nitrotyrosine antibody (Millipore, Billerica, MA) and mouse anti‐8‐hydroxy‐2‐deoxyguanosine (8‐OHdG) antibody (JaICA, Shizuoka, Japan) were used as markers of oxidative stress. Rabbit anti‐XO antibody (Abcam, Cambridge, MA), rabbit anti‐NADPH oxidase 4 (NOX4) antibody (NOVUS biologicals, Littleton, CO), rabbit anti‐rat manganese superoxide dismutase (SOD) (MnSOD; Enzo Life Sciences, Farmingdale, NY), and rabbit anti‐GAPDH antibody (Trevigen, Gaithersburg, MD) were also used.

### Immunohistochemistry

Methyl Carnoy's solution‐fixed, paraffin‐embedded sections were used for immunohistochemistry as described previously (Shiraishi et al. 2014). Briefly, after deparaffinization, the sections were incubated with primary antibodies for 1 h at 37°C. The sections were treated with 3% H_2_O_2_ for 10 min to inactivate endogenous peroxidase activity, followed by treatment with secondary antibodies for 1 h. The whole area of the kidney cortex, containing at least 20 glomeruli, was then examined. To determine the number of WT‐1‐positive cells, we examined both the glomeruli and the kidney cortex in this model. To assess the type IV collagen‐, podocin‐, and 8‐OHdG‐positive areas, the digital images at 200× magnification were analyzed using Image scope software. The percent positive area was determined as the diaminobenzidine‐positive pixels per total pixels in the glomerular tuft area in each section and averaged in all glomeruli.

### Western blotting

Kidney cortex on day 10 was homogenized in cell lysis buffer (Cell Signaling, Danvers, MA) at 4°C. Briefly, samples were processed for SDS‐PAGE and electro‐transferred onto a polyvinylidene fluoride membrane. After 1‐h incubation with a primary antibody at room temperature, the membrane was incubated with a secondary antibody linked with horseradish peroxidase for 1 h at room temperature. Signal was detected by SuperSignal West Pico Substrate (Thermo Fisher Scientific, Waltham, MA). The density of each band was determined using Multi Gauge software (Fujifilm, Tokyo, Japan) and expressed as a value relative to the density of the corresponding band of the GAPDH.

### Electron microscopy

The preparation for transmission electron microscopy (TEM) was performed according to standard procedures as described previously (Asakawa et al. 2017). The tissue blocks were fixed in 2.5% glutaraldehyde and embedded in Epon. Next, these samples were fixed using 2% osmium tetroxide, followed by dehydration with progressive concentrations of ethanol. Each sample was sectioned 0.6 *μ*m apart from each other. Samples from each glomerulus were studied using an H7520 electron microscope (Hitachi High‐Technologies Corporation, Tokyo, Japan). The analysis of the foot process of podocytes was performed at a magnification of 12 000.

### Cell culture

Immortalized mouse podocytes (Mundel et al. 1997) were cultured in DMEM/F‐12 medium (Gibco‐Invitrogen, Carlsbad, CA), supplemented with 10% fetal bovine serum, penicillin (100 U/mL), and streptomycin (100 *μ*g/mL). After induction of differentiation at 37°C for 2 weeks, subconfluent cells were stimulated with 12 mg/dL UA (Sigma, Saint Louis, MO) in serum‐free medium. Thirty minutes after UA exposure, NOX4 expressions in the cells were examined (*n* = 4).

### Statistical analysis

All values are expressed as mean ± SD. Comparison of continuous variables among groups more than 2 groups was performed by ANOVA, followed by Tukey's HSD post hoc test. Time‐dependent data were analyzed using repeated measures ANOVA, followed by Bonferroni multiple comparison test. Linear regression analysis was performed by Pearson product‐moment correlation. All statistical analyses were conducted using Graph Pad Prism 7 (GraphPad Software, San Diego, CA). A value of *P *<* *0.05 was considered statistically significant.

## Results

### Topiroxostat ameliorated serum total cholesterol and albumin levels and proteinuria in PAN rats

We examined the changes in body weight and kidney function after administration of topiroxostat (Table 1). On day 10, body weights of PAN rats did not increase so much as those without PAN irrespective of topiroxostat treatment. Since the body weights were greater than those on day 0, the rats did not lose weight but gained less weight due to eating less food and excreting more urine. Thus, it is suggested that PAN rats received less amounts of chow and topiroxostat as compared with those without PA. Urinary volume significantly increased only in PAN rats by ANOVA (Tukey HSD, *P *=* *0.009 vs. CONT), whereas no such increment was observed in PAN + Tx rats. PAN rats exhibited poor kidney function when compared with that of control rats, but not PAN + Tx rats, as evidenced by elevations of serum creatinine levels and declines in creatinine clearance (Ccr) on day 10. Total serum cholesterol level was significantly elevated in PAN rats while topiroxostat treatment significantly prevented this increase in PAN + Tx rats. Serum albumin levels declined in PAN rats, whereas topiroxostat treatment significantly prevented the decline in the levels of serum albumin in PAN + Tx rats.

**Table 1:** Laboratory data

	CONT (*N* = 6)	PAN (*N* = 5)	Tx (*N* = 6)	PAN + Tx (*N* = 6)
Body weight (g)				
0 day	175.5 ± 14.0	174.8 ± 12.6	173.2 ± 10.8	180.2 ± 11.1
10 days	252.0 ± 10.0^b^	200.2 ± 36.2	252.5 ± 9.9^b^	219.2 ± 10.5
Urinary volume (mL/day)				
0 day	5.7 ± 1.2	13.4 ± 9.1	11.2 ± 8.0	5.6 ± 1.7
10 days	5.2 ± 0.9^a^	20.4 ± 13.3	9.5 ± 4.9	12.7 ± 4.3
Creatinine clearance (mL/min/100 g)				
0 day	0.74 ± 0.13	0.79 ± 0.13	0.74 ± 0.12	0.69 ± 0.04
10 days	0.76 ± 0.12^b^	0.41 ± 0.08	0.84 ± 0.09^b^	0.45 ± 0.12
Serum creatinine (mg/dL)				
0 day	0.20 ± 0.02	0.22 ± 0.03	0.21 ± 0.02	0.20 ± 0.03
10 days	0.21 ± 0.03^b^	0.50 ± 0.17	0.21 ± 0.02^b^	0.46 ± 0.16
Serum total cholesterol (mg/dL)				
0 day	75.2 ± 15.2	74.6 ± 20.2	65.3 ± 12.2	82.8 ± 16.6
10 days	80.8 ± 7.8^b^	321.4 ± 40.1	76.3 ± 17.3^b^	235.2 ± 59.5^b^
Serum albumin (g/dL)				
0 day	3.80 ± 0.29	3.80 ± 0.19	3.65 ± 0.14	3.75 ± 0.24
10 days	4.02 ± 0.20^b^	2.12 ± 0.19	3.78 ± 0.19^b^	2.62 ± 0.37^a^
Hypoxanthine (mg/dL)				
10 days	Undetectable	Undetectable	0.14 ± 0.04	0.23 ± 0.14
Xanthine (mg/dL)				
10 days	Undetectable	Undetectable	0.24 ± 0.04	0.32 ± 0.09

PAN rats treated with topiroxostat. Data are expressed as mean ± SD. CONT, control; PAN, Puromycin aminonucleoside nephrosis rats; Tx, Control rats treated with topiroxostat; PAN + Tx, PAN with topiroxostat treatment group.

a *P *<* *0.01 versus PAN.

b *P *<* *0.001 versus PAN.

**Figure 1:**
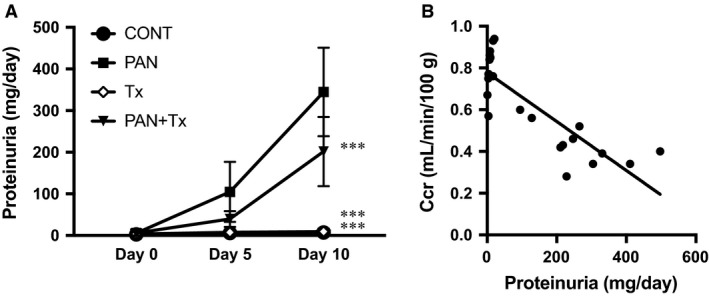
(A) Anti‐proteinuric effect of topiroxostat in PAN rat. Proteinuria was measured in 24‐h urine collection. CONT, control group; PAN, puromycin aminonucleoside nephrosis rats group; Tx, control rats treated with topiroxostat; PAN + Tx, PAN rats treated with topiroxostat. Data are expressed as mean ± SD. *** indicates *P *<* *0.001 versus PAN. (B) Relationship between proteinuria and creatinine clearance in all rats. Creatinine clearance on day 10 and proteinuria on day 10 are plotted. The regression line is *Y* = 0.78−0.00117*X* (*R* ^*2*^ 0.713, *P *<* *0.001). Ccr, creatinine clearance.

An increase in urinary protein excretion was evident in PAN rats than in control rats by repeated measures ANOVA. Topiroxostat significantly but not completely reduced urinary protein excretion on day 10 (Fig. 1A). The partial improvement by topiroxostat may be explained by the less intake of topiroxostat‐containing chow as suggested by less body weight, because Ccr on day 10 was correlated closely with urinary protein excretion on day 10 (Fig. 1B, *R* ^*2*^ 0.713, *P *<* *0.001), indicating inconsistent intake of oral topiroxostat despite the same dosage of injected PA. The result also suggested that kidney dysfunction seen in PAN rats is ascribed to the amount of proteinuria. Serum hypoxanthine and xanthine levels, both substrates for XO enzyme, were measured on day 10 to confirm whether topiroxostat worked or not. Serum hypoxanthine and xanthine levels were undetectable in control and PAN rats. In contrast, these substrates markedly increased in Tx rats and PAN + Tx rats (Table 1), indicating the effect of XO inhibition by topiroxostat.

### Effect of topiroxostat on kidney histology

**Figure 2:**
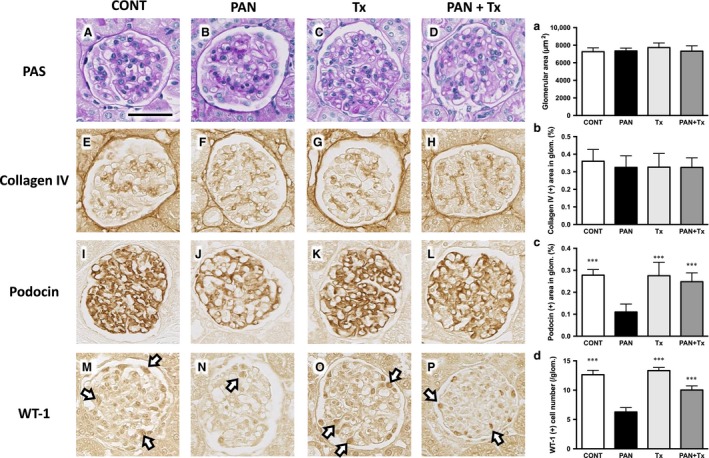
Topiroxostat ameliorates glomerular histopathology of PAN rats. Periodic acid‐Schiff (PAS) staining shows no change in glomerular size and pathology in PAN rats (B) when compared with that of CONT (A), Tx (C), and PAN + Tx (D). (a) quantification for glomerular size shows no significant difference. Glomerular collagen IV deposition (brown color) is prominent in PAN (E) than in CONT (F), Tx (G), and PAN + Tx (H). (b) quantification for collagen IV deposition shows no significant difference. Podocin (a slit membrane protein) and WT‐1 are reduced in PAN (J and N) than in CONT (I and M); however, PAN + Tx attenuates the downregulation of podocin expression and the loss of podocytes (L and P). (c) and (d) quantification for podocin and WT‐1, respectively. CONT, control group; PAN, puromycin aminonucleoside nephrosis rats group; Tx, control rats treated with topiroxostat; PAN + Tx, PAN rats treated with topiroxostat. Positive staining of podocytes are in part shown by arrows (M–P). Data are expressed as mean ± SD. *** indicates *P *<* *0.001 versus PAN. Scale bar in panel A indicates 50 *μ*m.

As shown in Figure 2, no significant difference in the glomerular size or collagen IV deposition was observed among all groups. However, PAN rats exhibited podocyte injury, as evidenced by a reduction in podocin expression and WT‐1 (+) cell number, and the intervention with topiroxostat significantly ameliorated such podocyte injuries.

**Figure 3:**
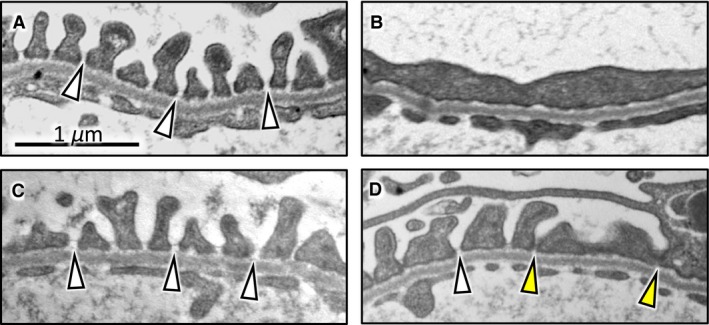
Transmission electron micrographs of podocyte foot process in the glomeruli. Normal appearance of glomerular basement membrane and filtration slits (white arrow head) are shown in CONT (A) and Tx (C). Foot process effacement is prominent in PAN rats (B). In PAN + Tx rats (D), the structure of foot processes looks relatively preserved (white arrow head) although some junctions between foot processes remain closed (yellow arrow head). CONT, control group; PAN, puromycin aminonucleoside nephrosis rats group; Tx, control rats treated with topiroxostat; PAN + Tx, PAN rats treated with topiroxostat. Scale bar in panel A indicates 1μm.

To further confirm the effect of topiroxostat in the kidney, ultrastructure of podocytes was examined using TEM. Figure 3A shows normal podocyte architecture in a control rat kidney; the integrity of such structures was maintained in the Tx rats (Fig. 3C). In the PAN rats, massive effacement of glomerular foot processes was uniformly observed (Fig. 3B). In PAN + Tx rats, the structure of foot processes looked relatively preserved although some junctions between foot processes were closed. The partial improvement of foot process effacement is also explained by the partial improvement of kidney dysfunction and proteinuria.

### Topiroxostat inhibited oxidative stress

**Figure 4:**
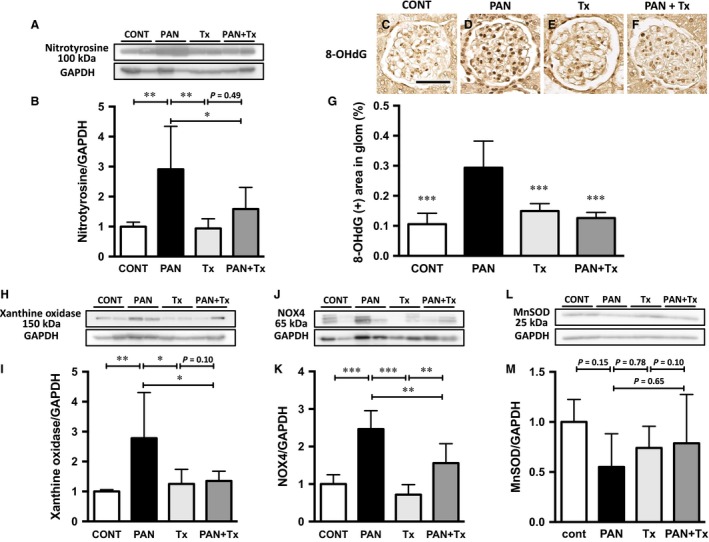
Topiroxostat reduces oxidative stress in PAN rats. (A) Western blot analysis of the protein lysate from the kidney cortex demonstrates that nitrotyrosine level is significantly higher in PAN rats than in CONT, and such an increase in nitrotyrosine level is significantly attenuated by topiroxostat. (B) Quantification of nitrotyrosine bands. (C) Glomerular 8‐hydroxy‐2‐deoxyguanosine (8‐OHdG) deposition (brown color) is more prominent in PAN rats (D) than in CONT (C), Tx (E), and PAN + Tx (F). (G) Quantification of intensity of immunohistochemical signal of 8‐OHdG. (H) Western blot analysis of the protein lysate from the kidney cortex shows that XO protein expression is higher in PAN rats than in CONT and that the upregulation of XO is attenuated by topiroxostat treatment. (I) Quantification of XO protein bands. (J) Western blot analysis of the protein lysate from the kidney cortex shows that NOX4 protein expression is higher in PAN rats than in CONT rats. The upregulation of NOX4 is attenuated by topiroxostat treatment. (K) Quantification of NOX4 protein bands. (L) Western blot analysis of the protein lysate from the kidney cortex reveals no change in manganese SOD (MnSOD) protein expression. (M) Quantification of MnSOD protein band shows no significant difference. CONT, control group; PAN, puromycin aminonucleoside nephrosis rats group; Tx, control rats treated with topiroxostat; PAN + Tx, PAN rats treated with topiroxostat. Data are expressed as mean ± SD. * indicates *P *<* *0.05 versus PAN, ** indicates *P *<* *0.01 versus PAN, and *** indicates *P *<* *0.001 versus PAN. Scale bar in panel C indicates 50 *μ*m.

To investigate the mechanisms of renoprotection, we first examined oxidative stress, a well‐known mediator of kidney disease. Nitrotyrosine levels in the kidney cortex were significantly higher in PAN rats than in control rats (Fig. 4A and B). However, this elevation was significantly inhibited by topiroxostat treatment (Fig. 4A and B). Similarly, the levels of 8‐OHdG, another marker of oxidative stress, were more prominent in PAN rats than in control rats, and were lowered by topiroxostat treatment (Fig. 4C–G). To further clarify the mechanisms by which topiroxostat blocks oxidative stress, the expressions of XO and NOX4 were examined. The protein levels of XO and NOX4 were observed to be significantly elevated in the PAN rat kidney when compared with those in the control rat kidney. Interestingly, topiroxostat significantly inhibited the elevation in protein levels of XO and NOX4 (Fig. 4H–K). It is apparently strange that XO expression was downregulated in response to XO inhibitor, the reason of which is likely conjectured by a speculation that accelerated degradation of the enzyme might occur following binding to the inhibitor in this setting. Of note is that several bands of NOX4 protein were detected probably due to the modification of the protein in murine (Mouche et al. 2007).

**Figure 5:**
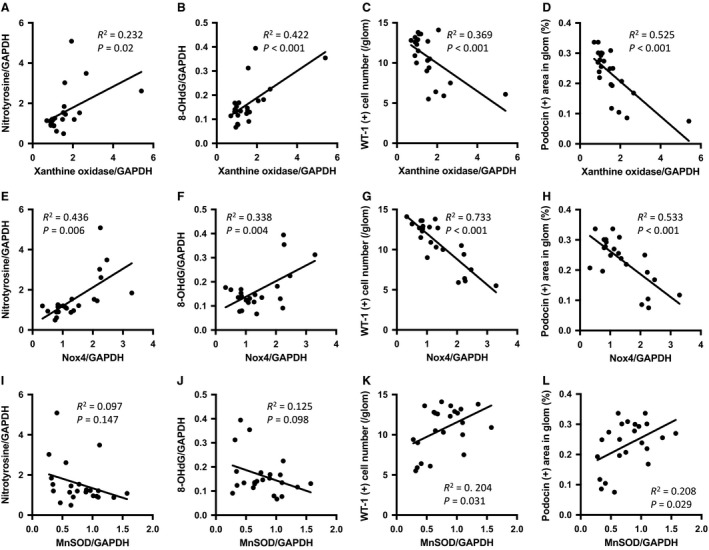
Correlations among oxidative stress markers. Correlations of levels of xanthine oxidase with those of nitrotyrosine and 8‐OHdG, WT‐1 (+) cell number, and podocin expression are shown (A–D). Correlations of levels of NOX4 with those of nitrotyrosine and 8‐OHdG, WT‐1 (+) cell number, and podocin expression are shown (E–H). Correlations of levels of manganese SOD (MnSOD) with those of nitrotyrosine and 8‐OHdG, WT‐1 (+) cell number, and podocin expression are shown (I–L). The coefficient of determination and *p* value are shown in each panel.

The expression levels of manganese superoxide dismutase (MnSOD) protein, an antioxidant marker, tended to be lower in PAN rat kidneys than in control kidneys, and its level tended to improve with topiroxostat treatment. However, the difference in MnSOD protein expression among the groups did not reach a statistical significance (Fig. 4L and M). Finally, XO and NOX4, which are known inducers of oxidative stress, were positively correlated with nitrotyrosine and 8‐OHdG levels in these rats. In contrast, MnSOD, an antioxidative enzyme, tended to be negatively correlated with the two oxidative stress markers – nitrotyrosine and 8‐OHdG. These data suggest that the mechanism for oxidative stress in this model could involve upregulation of XO and NOX4 expressions (Fig. 5A–L).

### Kidney and serum UA levels in PAN rats

**Figure 6:**
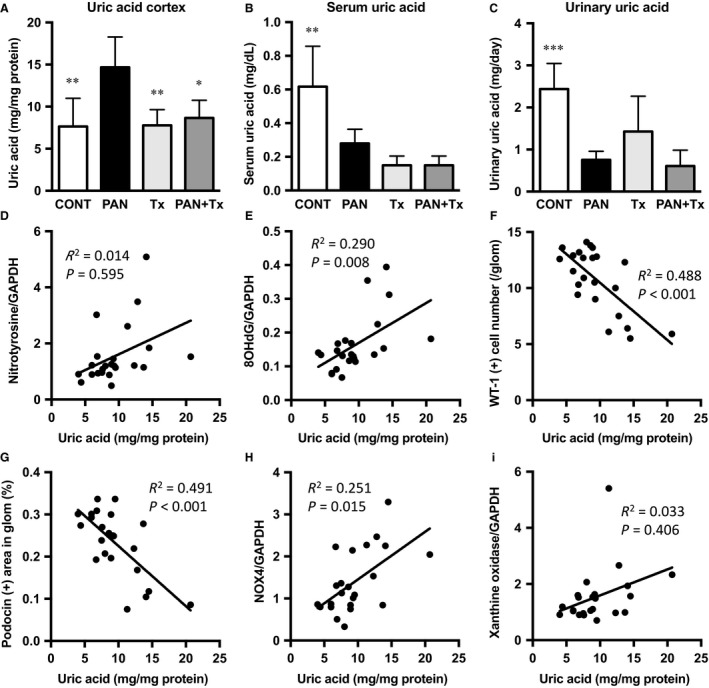
Intracellular uric acid and serum and urinary UA concentrations. The UA levels in the kidney cortex are significantly higher in PAN than in CONT, Tx, and PAN + Tx groups (A). Serum and urinary UA levels on day 10 are significantly lower in PAN rats than in CONT rats (B and C). Correlations of UA levels in kidney cortex with the expressions of nitrotyrosine and 8‐OHdG, WT‐1 (+) cell number, and podocin expression are shown (D–G). The expressions of NOX4 and xanthine oxidase are correlated with UA in the kidney cortex (H and I). The coefficient of determination and *P* value are shown in each panel. CONT, control group; PAN, puromycin aminonucleoside nephrosis rats group; Tx, control rats treated with topiroxostat; PAN + Tx, PAN rats treated with topiroxostat. Data are expressed as mean ± SD. * indicates *P* < 0.05 versus PAN and ** indicates *P* < 0.01 versus PAN.

The amount of UA in the kidney cortex of PAN rats was significantly higher when compared with those in control and Tx rats. However, topiroxostat treatment significantly reduced the UA amount in the kidney cortex of PAN rats (Fig. 6A). Of interest is that serum and urinary UA levels were significantly lower in PAN rats than in control rats, albeit UA in the kidney cortex was higher in PAN rats. This apparently strange result might be partly due to less intake of rat chow and an alternate possibility is debated in the Discussion part. Topiroxostat did not alter serum UA levels in PAN + Tx rats (Fig. 6B and C). Interestingly, the UA level in the kidney cortex was positively correlated with oxidative stress surrogate markers, nitrotyrosine and 8‐OHdG levels (Fig. 6D and E), suggesting that intracellular UA could contribute to an increase in oxidative stress in the PAN rats. In addition, the UA level in the kidney was negatively correlated with WT‐1 and podocin expressions (Fig. 6F and G), both podocyte injury markers. Furthermore, NOX4 expression in the kidney cortex positively increased with UA level in the same tissue (Fig. 6H), indicating that NOX4 upregulation may be an upstream event to render oxidative stress via generating superoxide in the PAN kidney cortex. Meanwhile, XO protein expression was not closely associated with the UA levels in the kidney cortex tissue (Fig. 6I), probably suggesting the complex phenomenon that PA‐induced XO activity leads to UA production in the cells, whereas intracellular UA once produced in turn inhibits XO activity. Moreover, XO activity is not directly linked with the expression of XO protein as mentioned.

### Uric acid‐induced expression of NOX4 in podocytes

**Figure 7:**
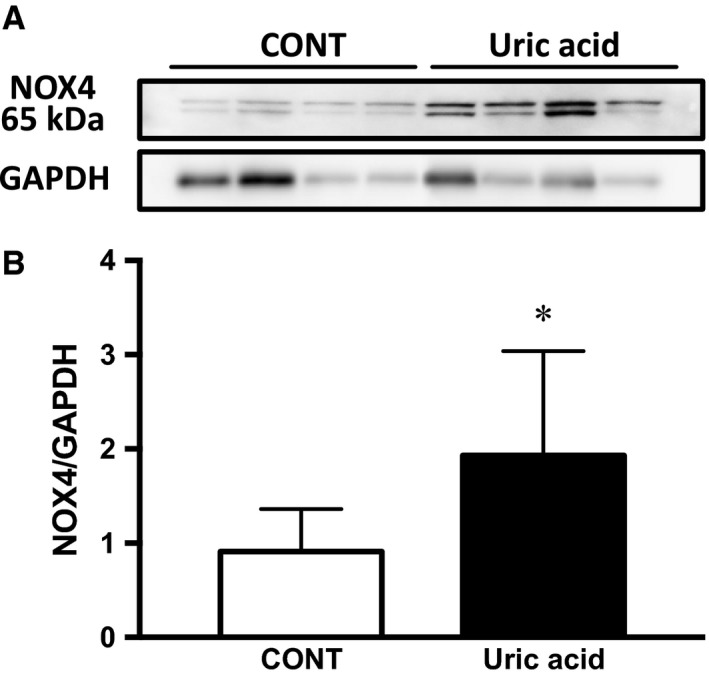
NOX4 expression in podocytes is induced by uric acid. Subconfluent mouse podocytes were stimulated with 12 mg/dL UA in serum‐free medium. Thirty minutes after UA exposure, NOX4 expression in the cells were significantly increased (*n* = 4). A) Several bans are seen probably due to the modification of the protein. B) Quantification of NOX4 protein bands. Data are expressed as mean ± SD. * indicates *P *<* *0.05 versus CONT.

Since the expression of XO and NOX4 were increased in PAN rats, we were prompted to examine whether NOX4 expression is dependent of XO activity or of intracellular UA level. To address this question, immortalized mouse podocytes were utilized after inducing differentiation of the cell function for 2 weeks in culture. NOX4 protein was significantly induced 30 min after adding UA of 12 mg/dL in the culture media (Fig. 7). Several bands of NOX4 protein were also detected in this murine cells (Mouche et al. 2007). This rapid response clearly showed that NOX4 expression is a downstream phenomenon of the increase in UA and independently of XO activity because hyperuricemia rather downregulates the XO activity as shown in the kidney cortex taken from the oxonic acid‐induced hyperuricemic model rats (Asakawa et al. 2017).

**Figure 8:**
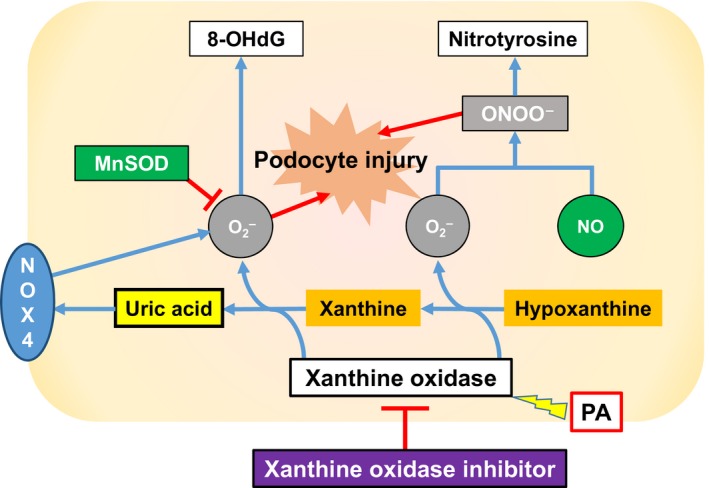
Schematic presentation of protective effects of topiroxostat in puromycin aminonucleoside nephrosis rats. Puromycin aminonucleoside activates xanthine oxidase in the cell, then generates superoxide free radical when catalyzing hypoxanthine to xanthine and to uric acid. These reactive oxygen species injure podocytes leading to foot process effacement and finally to massive proteinuria. Meanwhile, NOX4 expression is upregulated probably via intracellular UA accumulation rather than xanthine oxidase activity. Xanthine oxidase inhibitor, topiroxostat, attenuates these oxidative stress by inhibiting xanthine oxidase activity and its expression, resulting in the kidney protection. 8‐OHdG, 8‐hydroxy‐2‐deoxyguanosine; MnSOD, manganese superoxide dismutase; NOX4, NADPH oxidase 4; PA, puromycin aminonucleoside.

## Discussion

### PA‐induced nephrosis

PA administration in rats induces severe proteinuria and mimics the lesions of minimal change disease by single injection (Diamond et al. 1986; Inokuchi et al. 1996). PA‐induced nephrosis specifically leads to podocyte injury; foot process effacement, and decreased expression of slit diaphragm proteins such as nephrin and podocin, concomitant with the onset of proteinuria (Caulfield et al. 1976; Thakur et al. 1988; Lowenborg et al. 2000; Guan et al. 2004). Previous evidence indicates that reactive oxygen metabolites could be important mediators of tissue injury in PA‐induced nephrosis (Diamond et al. 1986; Thakur et al. 1988; Gwinner et al. 1997). The novel finding in this study is that topiroxostat inhibited the development of proteinuria and podocyte injury in PA‐induced nephrosis. Interestingly, this beneficial effect of topiroxostat was independent of serum UA levels. Rather, a lowering of UA concentration in the kidney cortex may account for the renoprotective effect of topiroxostat. In addition, the protective mechanism of topiroxostat could also be attributed to reduction in oxidative stress.

### Inhibiting proteinuria by topiroxostat

Previous studies demonstrated that topiroxostat reduced urinary albumin excretion in adenine‐induced kidney injury mice and in diabetic db/db mice (Kamijo‐Ikemori et al. 2016; Nakamura et al. 2016). In this study, we used PAN rats to examine whether topiroxostat could improve proteinuria and kidney function in a different type of kidney injury model. We found that topiroxostat exerts an anti‐proteinuric effect in PAN rats. Notably, topiroxostat protected podocytes from PA‐induced injury. Because superoxide free radical, one of the reactive oxygen species (ROS), induced by XO and NOX4 activity, likely leads to podocyte injury, the ability of topiroxostat to inhibit these enzymes could account for the protective effects of the drug on podocyte. In addition, our data suggest that the increased UA concentration in the kidney cortex is involved in podocyte injury because lowering UA level in the kidney cortex by topiroxostat was associated with podocyte protection. Based on these findings, targeting podocytes might be one of the mechanisms for the anti‐proteinuric effect of topiroxostat.

### Oxidative stress

To elucidate the protective effect of topiroxostat, we investigated the role of oxidative stress and its regulation in the kidney. We propose that the protective effect of topiroxostat can be ascribed to two mechanisms of reducing oxidative stress (Fig. 7). First is the inhibition of XO by topiroxostat. XO catalyzes the oxidation of hypoxanthine to xanthine and can further mediate the oxidation of xanthine to UA. These reactions produce superoxide free radical. Thus, the property of topiroxostat to inhibit XO can reduce oxidative stress in theory. As expected, we found that topiroxostat significantly reduced nitrotyrosine and 8‐OHdG, the two prominent markers of oxidative stress, and these findings were associated with a reduction in XO expression.

The other mechanism for topiroxostat protection likely involves inhibition of NOX4. In the kidney, ROS may be primarily produced by NOX4 (Gorin et al. 2005; Gill and Wilcox 2006; Sedeek et al. 2010). Five different isoforms of the NOX family have been identified, among which NOX1, NOX2, and NOX4 are present in the kidney cortex (Bedard and Krause 2007). NOX4 is the most abundant isoform in the kidney, and plays multiple roles following various stimuli (Sedeek et al. 2010). In cells expressing high levels of NOX4, the generation of large amounts of ROS is found to be responsible for cellular apoptosis (Zhu et al. 2013). High glucose‐induced NOX4 activation leads to cellular apoptosis both in cultured mouse podocytes and in diabetic mouse models (Susztak et al. 2006). In addition, NOX4‐mediated mitochondrial dysfunction is also involved in PA‐induced podocyte injury likely via increase in ROS generation (Yu et al. 2014). This study suggests that PA‐induced NOX4 activation leads to elevated oxidative stress and kidney disease. NOX4 may be involved in the signaling pathway downstream of tissue UA in PA‐induced podocyte damage (Lanaspa et al. 2014). Likewise, Verzola and associates reported that extracellular UA caused the increase in NOX4 expression and the induction of apoptosis in the renal tubule cell lines in culture which was abrogated by URAT1 inhibitors, probenecid and losartan (Verzola et al. 2014). In the present in vivo study, we cannot determine the underlying mechanism; thus, we have done an in vitro experiment using cultured podocytes by increasing UA concentration to 12 mg/dL to address this important question. The result showed that UA in the medium clearly increased NOX4 expression, suggesting that increased UA within the cell may be causative in increasing NOX4 expression independently of XO activity. This finding is analogous to our recent report by increasing serum UA by administration uricase inhibitor oxonic acid in the rats in which XO activity was downregulated by increase in intracellular UA (Asakawa et al. 2017). We need to elucidate the molecular mechanism on the linkage between cell UA and NOX4 activation in the future.

### Serum and urinary UA and intracellular UA

Because the serum UA level was significantly lower in PAN rats than in control rats and urinary UA level also decreased in the PAN rats, insufficient food intake was suspected. Accordingly, oral intake of topiroxostat was decreased than we expected before starting the experiments. However, this fact did not give rise to a big problem; rather resulted in the variation of the data, allowing us to examine the correlation analysis among them. Of particular note is that creatinine clearance on day 10 was closely correlated with proteinuria on day 10, suggesting that the main cause of kidney dysfunction is ascribed to the degree of proteinuria reflected by so high a coefficient of determination as 71%.

Despite the decline in serum UA concentration, UA level in the kidney cortex was markedly elevated in PAN rats, suggesting another key player of the mechanism of PA‐induced injury. It is known that UA, a metabolite of XO activation, is also a pro‐oxidant and may play a crucial role in the kidney disease progression (Nakagawa et al. 2006a, 2006b, 2006c, 2008). Hence, an elevation of intrarenal UA level, observed in this study, could further exacerbate oxidative stress probably via stimulation of NOX4. Since intracellular UA was found to be associated with podocyte injury as well as oxidative stress, UA might be a causal factor in the development of podocyte injury in this model. Schematic presentation of protective effects of topiroxostat in PAN rats is depicted in Figure 8.

## Conclusion

Topiroxostat ameliorates proteinuria and kidney injury independently of serum UA level in PAN rats. Topiroxostat exerts a renoprotective effect owing to its antioxidant effects and property to lower UA level in the kidney cortex.

## Conflict of interest

The authors declare no conflicts of interest.
